# The Predictive Values of Changes in Local and Remote Brain Functional Connectivity in Primary Angle-Closure Glaucoma Patients According to Support Vector Machine Analysis

**DOI:** 10.3389/fnhum.2022.910669

**Published:** 2022-05-19

**Authors:** Qiang Fu, Hui Liu, Yu Lin Zhong

**Affiliations:** ^1^Department of Emergency, Jiangxi Provincial People’s Hospital, The First Affiliated Hospital of Nanchang Medical College, Nanchang, China; ^2^Department of Ophthalmology, Jiangxi Provincial People’s Hospital, The First Affiliated Hospital of Nanchang Medical College, Nanchang, China

**Keywords:** primary angle-closure glaucoma, functional magnetic resonance imaging, regional homogeneity, functional connectivity, support vector machine

## Abstract

**Purpose:**

The primary angle-closure glaucoma (PACG) is an irreversible blinding eye disease in the world. Previous neuroimaging studies demonstrated that PACG patients were associated with cerebral changes. However, the effect of optic atrophy on local and remote brain functional connectivity in PACG patients remains unknown.

**Materials and Methods:**

In total, 23 patients with PACG and 23 well-matched Health Controls (HCs) were enrolled in our study and underwent resting-state functional magnetic resonance imaging (rs-fMRI) scanning. The regional homogeneity (ReHo) method and functional connectivity (FC) method were used to evaluate the local and remote brain functional connectivity. Moreover, support vector machine (SVM) method was applied to constructing PACG classification model.

**Results:**

Compared with the HC, PACG patients showed increased ReHo values in right cerebellum (CER)_8, left CER_4-5, and right CER_8. In contrast, PACG patients showed decreased ReHo values in the bilateral lingual gyrus (LING)/calcarine (CAL)/superior occipital gyrus (SOG) and right postcentral gyrus (PostCG). The ReHo value exhibited an accuracy of 91.30% and area under curve (AUC) of 0.95 for distinguishing the PACG patients from HC.

**Conclusion:**

Our study demonstrated that the PACG patients showed abnormal ReHo value in the cerebellum, visual cortex, and supplementary motor area, which might be reflect the neurological mechanisms underlying vision loss and eye pain in PACG patients. Moreover, the ReHo values can be used as a useful biomarker for distinguishing the PACG patients from HCs.

## Introduction

Glaucoma is the second leading cause of blindness globally; the number of patients with glaucoma is expected to increase to 79.6 million by 2020 (Quigley and Broman, 2006). Patients with glaucoma exhibit high intraocular pressure and optic atrophy. Glaucoma can be stratified into open-angle and closed-angle types. The risk factors for glaucoma include genetic (Wiggs and Pasquale, 2017) and environmental components (Lowe, 1972). Although medications and surgical procedures are the primary treatments for glaucoma, there is no effective treatment for advanced glaucoma. Neuroimaging studies have increasingly shown that the glaucoma causes optic nerve atrophy and damage to the visual pathway, including the visual cortex (Gupta et al., 2006; Gupta and Yucel, 2007).

The loss of retinal ganglion cells (RGCs) is the most important pathological change in PACG. The optic atrophy leads to transsynaptic neurodegenerative changes in visual pathways (Nucci et al., 2015; Mancino et al., 2018). Functional magnetic resonance imaging (fMRI) methods have been extensively used to detect abnormalities in brain structure and function in glaucoma patients. Wang et al. demonstrated that PACG patients had significantly lower fractional amplitude of low-frequency fluctuation (fALFF) values in the left cuneus, middle temporal gyrus, and right precentral gyrus; they had higher fALFF values in the bilateral superior frontal gyrus (Wang Q. et al., 2021). Chen et al. reported that the PACG patients showed increased short-range functional connectivity density (FCD) in the left inferior frontal gyrus (IFG)/insula/parahippocampal gyrus and right IFG/insula; they had decreased short-range FCD in the occipital lobe/cuneus/precuneus/superior parietal lobe/postcentral lobe (Chen et al., 2019). In an analysis of high-tension glaucoma patients, Wang et al. found the decreased functional connectivity (FC) within the visual network (Wang et al., 2020a). Moreover, Wang et al. demonstrated that primary open-angle glaucoma (POAG) patients had reduced cerebral blood flow (CBF)–functional connectivity strength (FCS) coupling and an altered CBF/FCS ratio in the visual cortex, salience network, default mode network (DMN), and dorsal attention network (Wang R. et al., 2021). Thus, glaucoma mainly causes abnormal function in the visual cortex. Additionally, glaucoma is accompanied by changes in brain structure. Wang et al. reported that high-tension glaucoma patients showed morphological reductions in visual and non-visual areas throughout the brain (Wang et al., 2020b). Furlanetto et al. reported that glaucoma patients had smaller optic nerve dimensions and shorter lateral geniculate nuclei compared to healthy controls (HCs) (Furlanetto et al., 2018). To our knowledge, the previous neuroimaging studies demonstrated that the glaucoma patients were accompanied by vision and vision-related brain region dysfunction. There have been few studies regarding the synchrony of neural activity changes in PACG patients. Thus, we assume that the PACG patients might be associated with abnormal local and remote brain functional connectivity.

The resting human brain exhibits homogeneous neural activity, which has an important role in visual function (Costa et al., 2017). Recently, fMRI has been used to investigate the changes in spontaneous neural activity in the human brain. Regional homogeneity (ReHo) analysis can be used to investigate the homogeneity of neural activity by measuring the functional coherence or synchronization of a particular voxel with its nearest voxels (Zang et al., 2004). ReHo analysis has revealed changes in the homogeneity of neural activity in vision-related diseases such as iridocyclitis (Tong et al., 2021) and dysthyroid optic neuropathy (Jiang et al., 2021). In addition to its high spatial and temporal resolution, ReHo analysis provides robust repeatability. In contrast to other fMRI technologies, ReHo method is data-driven technology without preliminary assumption. Moreover, the ReHo method can reflect the neural activity of the whole brain. Thus, ReHo analysis is suitable for non-invasive exploration of changes in neural activity within the brain. In recent years, the combined development of MRI and machine learning technology has provided powerful diagnostic tools. MRI-related machine learning has been successfully applied for accurate clinical classification and diagnosis of many diseases (Han et al., 2019; Niu et al., 2021). Support vector machine (SVM) is a popular tool for fMRI data analysis. SVM can provide a unique solution with a good out-of-sample generalization and an implicit implementation of non-linear classification using the kernel technique. SVM has been used to perform supervised classifications of specific brain functional states/disorders in a variety of task conditions. However, to our knowledge, there have been few studies concerning MRI-related machine learning in PACG patients.

Thus, the present study was performed to investigate the changes in the homogeneity of neural activity in PACG patients. We have investigated the changes in remote FC in PACG patients by region of interest (ROI) analysis by means of the ReHo technique. Moreover, a SVM method was used to determine whether aberrant ReHo could reliably distinguish PACG patients from HCs.

## Materials and Methods

### Participants

In total, 23 patients with PACG (10 men and 13 women) were enrolled from the Department of Ophthalmology, Jiangxi Provincial People’s Hospital. The diagnostic criteria of PACG were: (1) the intraocular pressure is greater than 21 mmHg in both eyes; (2) optic disc/cup area > 0.6; (3) typical vision field defect (paracentric obscura, arcuate obscura, nasal ladder, fan-shaped field defect, and peripheral field defect); (4) without any other ocular diseases (high myopia, optic neuritis, strabismus, amblyopia, cataracts, and retinal degeneration). The diagnosis of PACG was conducted by two experienced ophthalmologists. All glaucoma patients underwent anterior chamber angioscopy to make sure the front corner is open. The exclusion criteria of PACG were: (1) advanced PACG patients are associated with severe eye pain; (2) PACG patients with history of surgery; (3) PACG patients with glaucoma-related eye complications such as neovascular glaucoma POAG, secondary glaucoma cataracts, eye atrophy, and corneal edema.

Twenty-three patients’ healthy controls (HCs) (10 men and 13 women) were also recruited for this study. The inclusion criteria: (1) without any ocular disease with uncorrected visual acuity (VA) > 1.0; (2) no cardiovascular system diseases and no psychiatric disorders.

### Ethical Statement

All research methods were followed the Declaration of Helsinki and were approved by the Ethical Committee for Medicine of Jiangxi Provincial People’s Hospital. Participants were enrolled in the study of their own accord and were informed of the purpose, methods, as well as potential risks before signing an informed consent form.

### Clinical Evaluation

The VA of all subjects was measured using the logMAR table and intraocular pressure was assessed by the automatic intraocular pressure measurement. The best-corrected VA and intraocular pressure of both eyes were measured in the each group.

### Magnetic Resonance Imaging Data Acquisition

The MRI scanning was performed on a 3-Tesla MR scanner (750W GE Healthcare, Milwaukee, WI, United States) with an eight-channel head coil. All participants were required to close their eyes without falling asleep when undergoing MRI scanning. The subjects should be keep calm and not engage in specific thoughts. The T1 parameters (repetition time = 8.5 ms, echo time = 3.3 ms, thickness = 1.0 mm, gap = 0 mm, acquisition matrix = 256 × 256, field of view = 240 × 240 mm^2^, and flip angle = 12°) and 240 functional images parameters (repetition time = 2,000 ms, echo time = 25 ms, thickness = 3.0 mm, gap = 1.2 mm, acquisition matrix = 64 × 64, field of view = 240 × 240 mm^2^, flip angle = 90°, voxel size = 3.6 × 3.6 × 3.6 mm^3^, and 35 axial slices) covering the whole brain were obtained.

### Data Pre-processing

All preprocessing was performed using the toolbox for Data Processing and Analysis of Brain Imaging (DPABI^1^) (Yan et al., 2016) and briefly following the steps: (1) The first 10 volumes of each subject were removed. (2) Slice timing and head motion correction were conducted. (3) Normalized fMRI data were re-sliced with a resolution of 3 × 3 × 3 mm^3^. (4) Data detrends. (5) Linear regression analysis was applied to regress out several covariates (mean frame-wise displacement, global brain signal, and averaged signal from cerebrospinal fluid and white matter). (6) Temporal band-pass filtering was performed (0.01–0.08 Hz).

### Regional Homogeneity Analysis

The ReHo index was calculated by the DPABI software. All ReHo maps of each voxel were *z*-transformed with Fisher’s *r*-to-*z* transformation to reduce the influence of individual variation for group statistical comparisons.

### Functional Connectivity Analysis

The seed-based FC was used to investigate the remote FC ROI in different ReHo-related brain regions between two groups. Then we calculated the Pearson’s correlation coefficient between the representative time series of each seed ROI and every other voxel in the whole brain in the voxel-wise method. Finally, the generated correlations-coefficient maps were converted to *Z* values with Fisher’ *r*-to-*z* transformation to reduce the impacts of individual variations for group statistical comparisons.

### Support Vector Machine Analysis

The SVM algorithm was performed using the Pattern Recognition for Neuroimaging Toolbox (PRoNTo) software Cyclotron Research Centre, University of Liège, Belgium (Schrouff et al., 2013). The following steps were followed: (1) dataset specification, (2) the ReHo maps served as a classification feature, (3) Then, the leave-one-out cross-validation (LOOCV) technique was applied to perform SVM classifier validation for model estimation. In each LOOCV fold, FC data from *n* – 1 samples were selected as the training dataset to train the classification model. (4) The total accuracy, specificity, sensitivity, and area under the receiver operating characteristic curve (AUC) were calculated.

### Statistical Analysis

The independent sample *t*-test was used to investigate the clinical features between the two groups.

The one-sample *t*-test was conducted to assess the group mean of ReHo maps. The two-sample *t*-test was used to compare the two group differences in the ReHo and FC maps using the Gaussian random field (GRF) method (two-tailed, voxel-level *p* < 0.01, GRF correction, and cluster-level *p* < 0.05).

## Results

### Demographics and Disease Characteristics

There were no statistically significant differences between the PACG and HC groups in gender (*p* > 0.999), education (*p* = 0.506), or age (*p* = 0470), but significant differences in BCVA of right eye (*p* < 0.001) and left eye (*p* < 0.001). The results of these data were listed in Table 1.

**TABLE 1:** Demographic and clinical measurements between patients with primary angle-closure glaucoma (PACG) and healthy controls (HCs).

Condition	PACG group	HC group	*T*-value	*P*-value
Age (years)	50.96 ± 4.85	50.82 ± 6.76	0.727	0.470
Sex (male/female)	10/13	10/13	N/A	>0.999
Education (years)	12.61 ± 5.88	11.46 ± 6.86	0.670	0.506
BCVA-OD	0.15 ± 0.10	1.18 ± 0.12	0.626	<0.001
BCVA-OS	0.30 ± -0.12	1.14 ± 0.10	0.538	<0.001
Handedness	23 R	23 R	N/A	N/A

*Chi-square test for sex. Independent t-test was used for other normally distributed continuous data. Data are displayed as mean ± standard deviation. HC, healthy control; BCVA, best-corrected visual acuity; OD, oculus dexter; OS, oculus sinister; N/A, not applicable; R, right.*

### Comparisons of Regional Homogeneity Between Patients With Primary Angle-Closure Glaucoma and Health Control

**FIGURE 1:**
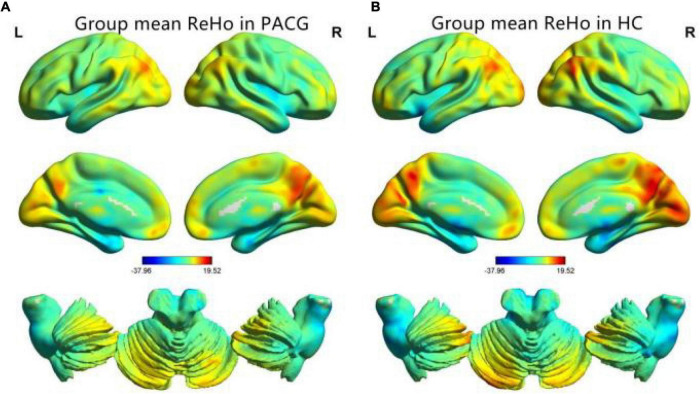
One-sample *t*-test results of regional homogeneity (ReHo) maps within the primary angle-closure glaucoma (PACG) **(A)** and One-sample *t*-test results of ReHo maps within the healthy control (HC) **(B)**. ReHo, regional homogeneity; PACG, primary angle-closure glaucoma; HC, healthy control.

**FIGURE 2:**
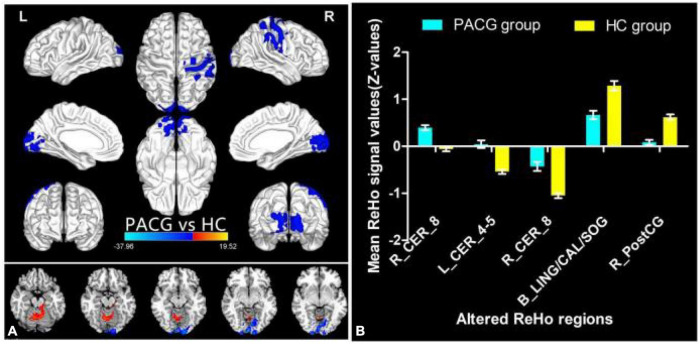
Different ReHo between the PACG and HC group. The red denotes higher ReHo values, and the blue areas indicate lower ReHo values (voxel-level *p* < 0.01, GRF correction, cluster-level *p* < 0.05) **(A)**. The mean of altered ReHo values between patients with PACG and HCs **(B)**. ReHo, regional homogeneity; PACG, primary angle-closure glaucoma; HC, healthy control; CER, cerebellum; LING, lingual gyrus; CAL, calcarine; SOG, superior occipital gyrus; PostCG, postcentral gyrus.

The group means of ReHo maps of the PACG and HC are shown in Figures 1A,B. Compared with the HCs, PACG patients showed significantly increased ReHo values in the right cerebellum (CER)_8 and left CER_4-5. Meanwhile, PACG patients showed decreased ReHo values in the bilateral lingual gyrus (LING)/calcarine (CAL)/superior occipital gyrus (SOG) and right postcentral gyrus (PostCG) (Table 2 and Figure 2A). The mean values of altered ReHo values were shown with a histogram (Figure 2B).

**TABLE 2:** Significant differences in the regional homogeneity (ReHo) between two groups.

		MNI				
Condition	Brain regions	*x*	*y*	*z*	Peak *T*-scores	Cluster size (voxels)
PACG > HC	R_CER_8	−12	−60	−63	6.4017	645
PACG > HC	L_CER_4-5	−12	−30	−27	4.5909	539
PACG > HC	R_CER_8	15	−21	−51	5.6261	143
PACG < HC	B_LING/CAL/SOG	−9	−102	18	−4.5399	738
PACG < HC	R_PostCG	39	−36	66	−4.7791	514

*x, y, and z are the locations of the peak voxels in standard MNI coordinates. ReHo, regional homogeneity; PACG, primary angle-closure glaucoma; HC, healthy control; MNI, Montreal Neurological Institute; CER, Cerebellum; LING, Lingual Gyrus; CAL, Calcarine; SOG, Superior Occipital Gyrus; PostCG, Postcentral Gyrus;R, right; L, left; B, bilateral.*

### Differences in Functional Connectivity

**FIGURE 3:**
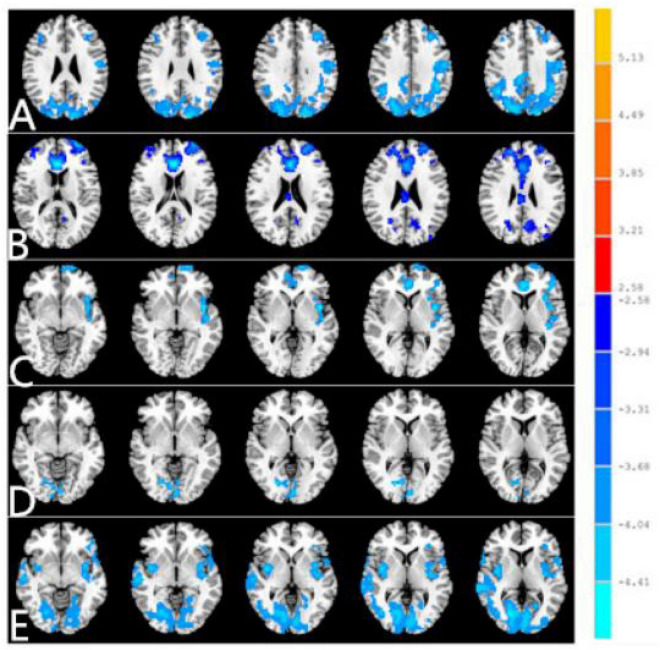
Different FC between two groups with region of interest (ROI) in R_CER_8 **(A)**. Different functional connectivity (FC) between two groups with ROI in L_CER_4-5 **(B)**. Different FC between two groups with ROI in R_CER_8. **(C)** Different FC between two groups with ROI in B_LING/CAL/SOG **(D)**. Different FC between two groups with ROI in R_PostCG **(E)**. The red denotes higher FC signal values, and the blue areas indicate lower FC signal values (voxel-level *p* < 0.01, GRF correction, cluster-level *p* < 0.01). FC, functional connectivity; PACG, primary angle-closure glaucoma; HC, healthy control; CER, cerebellum; LING, lingual gyrus; CAL, calcarine; SOG, superior occipital gyrus; PostCG, postcentral gyrus.

The seed-based FC was used to investigate the remote FC between two groups with ROI in the five different ReHo brain regions: (1) ROI in R_CER_8, the PACG patients showed decreased FC between the R_CER_8 and R_IPL, L_IFG, R_SFG (Table 3 and Figure 3A). (2) ROI in L_CER_4-5, the PACG patients showed decreased FC between the L_CER_4-5 and R_CER_8, R_CER_4_5, L_MFG, R_MFG, L_PreCUN (Table 3 and Figure 3B). (3) ROI in R_CER_8, the PACG patients showed decreased FC between the L_INS, and L_ACC, R_ANG, L_PreCUN (Table 3 and Figure 3C). (4) ROI in B_LING/CAL/SOG, the PACG patients showed decreased FC between the B_LING/CAL/SOG and R_CER, L_CAL, R_PostCG (Table 3 and Figure 3D). (5) ROI in R_PostCG, the PACG patients showed decreased FC between the R_PostCG and L_STG, L_INS, L_IFG, R_CAL, L_IFG (Table 3 and Figure 3E).

**TABLE 3:** Significant differences in the functional connectivity (FC) between two groups.

		MNI				
Condition	Brain regions	*x*	*y*	*z*	Peak *T*-scores	Cluster size (voxels)
ROI in R_CER_8						
PACG < HC	R_IPL	45	−54	42	−5.3942	7070
PACG < HC	L_IFG	−51	39	−9	−3.7121	196
PACG < HC	R_SFG	18	−3	57	–4.2058	485
ROI in L_CER_4-5						
PACG < HC	R_CER_8	15	−54	−60	−4.858	1990
PACG > HC	R_CER_4_5	15	–21	−24	5.1884	653
PACG < HC	L_MFG	−39	60	12	−4.5478	1076
PACG < HC	R_MFG	39	45	15	−3.2698	57
PACG < HC	L_PreCUN	−9	−66	42	−5.0912	4947
ROI in R_CER_8						
PACG < HC	L_INS	–42	3	0	–4.4446	723
PACG < HC	L_ACC	3	39	9	–4.1039	652
PACG < HC	R_ANG	21	–60	45	–4.4087	477
PACG < HC	L_PreCUN	–12	–63	39	–4.7861	988
ROI in B_LING/CAL/SOG						
PACG < HC	R_CER	18	–39	–60	–4.3662	469
PACG < HC	L_CAL	18	–96	–15	–3.3303	171
PACG < HC	R_PostCG	30	–33	54	–4.4334	4396
ROI in R_PostCG						
PACG < HC	L_STG	-30	15	–36	–3.6386	44
PACG < HC	L_INS	–36	–15	–12	–4.5824	593
PACG < HC	L_IFG	–51	39	–9	–3.5894	73
PACG < HC	R_CAL	18	–78	9	–5.1517	6779
PACG < HC	L_IFG	–33	33	3	–3.4947	48

*x, y, and z are the locations of the peak voxels in standard MNI coordinates. FC, functional connectivity; PACG, primary angle-closure glaucoma; HC, healthy control; MNI, Montreal Neurological Institute; CER, Cerebellum; LING, Lingual Gyrus; CAL, Calcarine; SOG, Superior Occipital Gyrus; PostCG, Postcentral Gyrus; IPL, Inferior Parietal Lobule; IFG, Inferior Frontal Gyrus; SFG, Superior Frontal Gyrus; MFG, Middle Frontal Gyrus; PreCUN, Precuneus; INS, Insula; ACC, Anterior Cingulate Gyrus; ANG, Angular Gyrus; PostCG, Precentral Gyrus; STG, Superior Temporal Gyrus; IFG, Inferior Frontal Gyrus; R, right; L, left; B, bilateral.*

### Support Vector Machine Results

**FIGURE 4:**
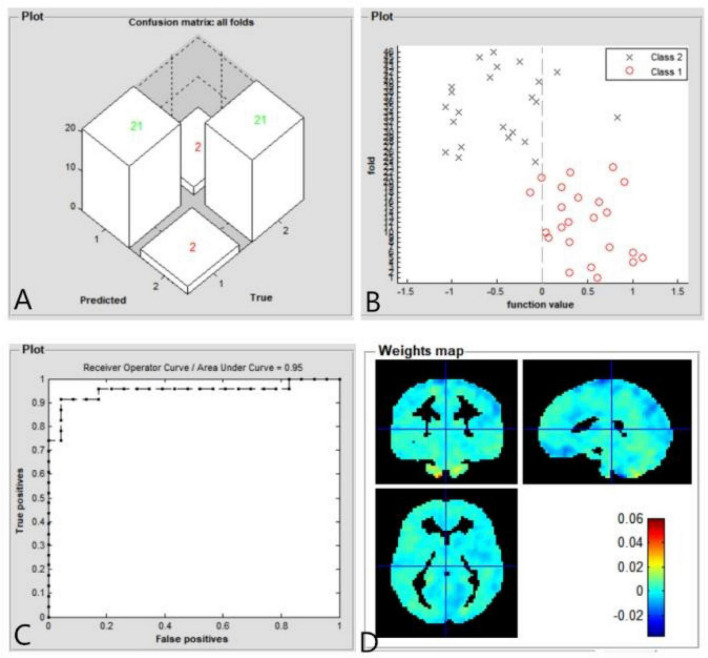
Classification results using machine learning analysis based on ReHo values. Three-dimensional confusion matrices from machine learning analysis **(A)**. Function values of two groups (class 1: PACG group; class 2: HC group) **(B)**. The ROC curve of the SVM classifier with an AUC value of 0.95 **(C)**. Weight maps for SVM models. The weight in each voxel corresponding to its contribution to the model’s prediction **(D)**.

The SVM classification reaches a total accuracy of 91.30%. Classification results using machine learning analysis were based on the ReHo values. Three-dimensional confusion matrices from machine learning analysis (Figure 4A). Function values of two groups (class 1: PACG group; class 2: HC group) (Figure 4B). The ROC curve of the SVM classifier with an AUC value of 0.95 (Figure 4C). Weight maps for SVM models. The weight in each voxel corresponding to its contribution to the model’s prediction (Figure 4D).

## Discussion

In this study, compared with HCs, PACG patients had significantly greater ReHo values in the right cerebellum (CER)_8 and left CER_4-5. PACG patients also showed decreased ReHo values in the bilateral lingual gyrus (LING)/calcarine (CAL)/SOG and right PostCG. Moreover, PACG patients showed abnormal remote FC within the cerebellar network, visual network, DMN, and sensorimotor network (SMN). Notably, the SVM classifier exhibited overall accuracy of 91.30% and an AUC of 0.95 based on ReHo values.

As noted above, PACG patients had significantly greater ReHo values in the right CER_8 and left CER_4-5, compared with the values in HCs. The cerebellum has important roles in motor control and body balance (Doya, 2000; Morton and Bastian, 2004), as well as higher cognitive function (Middleton and Strick, 1994). The cerebellum also has important roles in visual attention and working memory (Dai et al., 2013; Brissenden et al., 2018; van Es et al., 2019). Thus, the cerebellum participates in visual information processing. Dai et al. demonstrated that POAG patients had increased FC between V1 and the cerebellum (Dai et al., 2013). Song et al. also reported that POAG patients had increased ReHo values in the cerebellum (Song et al., 2014). In the present study, we found that increased ReHo in the cerebellum of PACG patients, which might reflect impaired visual attention in such patients.

Importantly, PACG patients had decreased ReHo values in the bilateral LING/CAL/SOG, which is located in the primary visual cortex. Furthermore, we found that PACG patients had decreased remote FC between the visual cortex and the CER, CAL, and PostCG. High intraocular pressure in PACG patients can lead to RGC loss, which results in decreased transmission of visual signals into the visual cortex. Furthermore, the previous neuroimaging studies demonstrated the visual pathway atrophy in glaucoma patients (Hernowo et al., 2011; Haykal et al., 2021). Other studies demonstrated that PACG patients had abnormal cortical thickness in the visual cortex (Yu et al., 2013; Bogorodzki et al., 2014). Thus, we found that the ReHo was decreased in the primary visual cortex of PACG patients, which might indicate abnormal visual information processing in the visual cortex.

In this study, we found that PACG patients had decreased ReHo values in the right PostCG. The PostCG has an important role in sensorimotor function (Zlatkina et al., 2016). The PostCG dysfunction might induce motor control impairment (Kato and Izumiyama, 2015). Vivek Trivedi et al. reported that visuomotor coordination might be impaired in glaucoma patients (Trivedi et al., 2019). Furthermore, the visual field defect might lead to slow movement and falls in PACG patients; glaucoma patients showed impairments in both visual and somatosensory function, compared to HCs (Black et al., 2008; Kotecha et al., 2012). Consistent with these findings, our study demonstrated that the PACG patients had decreased ReHo values in the PostCG, which might reflect sensorimotor dysfunction. Additionally, the PostCG is important for pain sensory processing. In our study, PACG patients showed symptoms of acute eye pain. The PostCG is also involved in pain information processing. The previous neuroimaging studies demonstrated that the eye pain-related diseases might induce PostCG dysfunction (Tang et al., 2018). Thus, we speculate that the impaired visuomotor coordination and acute eye pain contribute to PostCG dysfunction in PACG patients.

Finally, a machine learning technique (i.e., an SVM classifier) was used to determine whether aberrant ReHo values could reliably distinguish PACG patients from HCs. In our study, the SVM classifier accuracy was 91.30%. The ROC curve of the SVM classifier revealed an AUC of 0.95. Our findings indicate that the ReHo values can be used to reliably distinguish PACG patients from HCs. Therefore, the combination of ReHo analysis and machine learning can be used for disease classification and diagnosis, support the use of these methods in future clinical practice. Thus, SVM model showed high sensitive classification and diagnosis ability using ReHo as a feature. In the future, SVM model could be used for early diagnosis of glaucoma.

## Limitations

First, the sample size was small. Second, ReHo and FC values on the basis of blood oxygenation level-dependent signals would still be affected by physiological noise, such as cardiac and respiratory activity and scanning noise. Thirds, PACG patients have an inconsistent course of disease, which might be bad impact on the results of the study.

## Conclusion

Our results showed that PACG patients had abnormal homogeneity of neural activities such as cerebellum, visual cortex, and sensorimotor cortex, which might indicate the neural mechanism of visual field defect in patients with PACG. Moreover, ReHo map could be sensitive biomarkers for distinguishing patients with PACG from HCs.

## Data Availability Statement

The raw data supporting the conclusions of this article will be made available by the authors, without undue reservation.

## Ethics Statement

The studies involving human participants were reviewed and approved by Ethical Committee for Medicine of Jiangxi Provincial People’s Hospital. The patients/participants provided their written informed consent to participate in this study.

## Author Contributions

QF, HL, and YZ contributed to data collection, statistical analyses, wrote the manuscript, designed the protocol, MRI analysis, designed the study, oversaw all clinical aspects of study conduct, and manuscript preparation. All authors contributed to the article and approved the submitted version.

## Conflict of Interest

The authors declare that the research was conducted in the absence of any commercial or financial relationships that could be construed as a potential conflict of interest.

## Publisher’s Note

All claims expressed in this article are solely those of the authors and do not necessarily represent those of their affiliated organizations, or those of the publisher, the editors and the reviewers. Any product that may be evaluated in this article, or claim that may be made by its manufacturer, is not guaranteed or endorsed by the publisher.

## Footnotes

1
http://www.rfmri.org/dpabi
